# Excision of the Knee-joint

**Published:** 1853

**Authors:** 


*Excision of the Knee-joint.*

Mr. Smith introduced at a meeting of the Medical Society of London (November 19, 1853) for the inspection of the fellows, two persons on whom this operation of the excision of the knee-joint has been successfully performed by Mr. Jones, of Jersey, who was present at the meeting, and who had been induced at his request to allow them to be brought here. All those present must be aware that much discussion had been carried on respecting this operation, and that a great deal of opposition to it had arisen in certain quarters, and unfortunately we had not had sufficient experience in London to lead us to form a definite conclusion respecting the merits of the proceeding in question. In other parts of Great Britain, however, it had been adopted with great success, and especially in Jersey, by Mr. Jones, who had had six cases, five out of which were now quite well. Two of these patients were now presented. One of them, a young man of twenty, walked up the room without any artificial appliance whatever, and was able to use his limb very extensively, it being perfectly straight, and only half an inch shorter than its fellow, so that it is not necessary for him to wear a high-heeled boot. The knee is anchylosed, there being hardly any movement. The man is in perfect health, and stated that he had walked as far as six miles together, and is now enabled to carry on his occupation, which is that of a house-painter. The other was a boy, aged twelve, on whom the operation of excision had been performed seven months only. The limb was quite straight, as in the other, and there was perfect bony anchylosis at the knee. In this instance, however, the patella had been left so that the boy had full power of lifting the limb, inasmuch as the attachment of the great extensor tendon was left. It was noticed that this little boy walked up the room with the aid of two sticks. This was explained by the existence of a circumstance which rendered the case very interesting. This was a dislocation of the hip of the opposite side, which had occurred spontaneously from disease some few weeks after the operation. Fortunately, however, the disease in the hip had become arrested, and the patient, although he ordinarily used two sticks, was enabled to walk, resting upon the arm of another person. He was daily getting strength, and doubtless in time would be able to progress with facility. If, however, amputation of the thigh had been done, and he used a wooden leg, the dislocation of the hip on the other side would have prevented progression, the superiority, therefore, of excision of the knee-joint was doubly shown in this example.

Mr. Jones had operated in six cases; in five of these the operation was successful. The sixth would have been equally satisfactory, but the patient, a lady, died ten days after the operation, of dysentery, which was then prevalent, One of the patients he had operated upon could run well up a ladder, and another play at foot-ball. He had brought the two patients just exhibited up to London, to seek the opinion of the more experienced surgeons here, with a view to determine whether the operation were a legitimate one, or should be abandoned.

Lancet, November 23, 1853.

